# Supplementary material for: Hub connectivity, neuronal diversity, and gene expression in the Caenorhabditis elegans connectome
Source: PLoS Comput Biol. 2018 Feb 12;14(2):e1005989. doi: 10.1371/journal.pcbi.1005989 (PMC5825174; doi:10.1371/journal.pcbi.1005989)
Supplement: S1 Text — (PDF) [file pcbi.1005989.s001.pdf]

Expression annotations at WormBase

Neuronal gene expression is measured as a binary indicator on WormBase [63], based on curated data collated from many individual experiments. Expression annotations are made either 'directly' to individual neurons (when an experiment indicates expression in an individual neuron), or 'indirectly' to broader classes of neurons like 'interneuron' or 'head' (meaning that some members of that class exhibit expression of that gene). In order to maintain specificity of annotations, we only analysed 'direct' annotations here.

Annotations of gene *G* to neuron *Y* are also made with varying levels of certainty:

- 'certain': *G* was observed to be expressed in *Y*,
- 'enriched': *G* has been found to be enriched in a certain dataset through microarray, RNAseq or proteomic analysis,
- 'partial': *G* was observed to be expressed in some cells of a group of neurons that include Y,
- 'blank': data prior to 2005, or
- 'uncertain': *G* was sometimes observed to be expressed in *Y*, or *G* was observed to be expressed in a cell that could be *Y*.

To avoid including false positives, our analysis excluded annotations labeled as 'uncertain'.
