# Supplementary material for: Hub connectivity, neuronal diversity, and gene expression in the Caenorhabditis elegans connectome
Source: PLoS Comput Biol. 2018 Feb 12;14(2):e1005989. doi: 10.1371/journal.pcbi.1005989 (PMC5825174; doi:10.1371/journal.pcbi.1005989)
Supplement: S2 Text — (PDF) [file pcbi.1005989.s002.pdf]

Sensitivity of correlated gene expression measures

Measures of correlation between binary vectors can be biased by the relative proportion of ones between two vectors. For data analysed here, data annotated to individual neurons comes from between 3 to 138 expressed genes (corresponding to 0.3% to 14.6% of all 948 genes considered). To ensure that our measure of correlated gene expression (CGE) is not biased by such differences in the relative proportions of expression annotations, we conducted a sensitivity analysis in which we compared the r_ϕ metric, Eq. (3), with alternative methods for quantifying correlations between binary vectors: the Jaccard index, $n_{11}/(n_{10}+n_{01}+n_{11})$, Yule's Q coefficient, $(n_{00}n_{11}-n_{01}n_{10})/(n_{00}n_{11}+n_{01}n_{10})$, and the χ^2, index, $N(n_{00}n_{11}-n_{01}n_{10})/(n_{0\bullet}n_{1\bullet}-n_{\bullet 1}n_{\bullet 0})$ [60], where n_{xy} counts the number of observations of each of the four binary pairwise possibilities: n_{00}, n_{01}, n_{10}, and n_{11} (as outlined in the main text), and the • symbol sums across a given variable (e.g., $n_{\bullet}= n_{00} + n_{10}$).

To evaluate bias in each CGE measure to the proportion of annotations in each expression vector, we generated random binary vectors of length 948 containing different proportions of 1s seen in our data, ranging from the minimum, 1, to the maximum, 150. For all pairwise combinations of proportions, we computed the CGE measure, taking an average across 1 000 permutations, and then recorded the resulting mean correlation value, as plotted in S2 Fig. Because all vectors are independent random binary strings, any systematic dependence of mean CGE with annotation proportion indicates bias. The mean square contingency coefficient, r_ϕ (S2 FigA) and our own novel CGE matching index, p_{match} (S2 FigD) show no systematic dependence on the proportion of ones in each vector (varying randomly within $\approx 10^{-3}$ and ≈ 0.5 respectively). However, Yule's Q shows a negative bias for small annotation proportions (S2 FigB) and the Jaccard index shows a strong positive bias across the full range (S2 FigC). Based on these numerical experiments, we selected mean square contingency coefficient, r_ϕ, here, to ensure that changes in CGE were due to matching expression patterns and not simply driven by differences in the number of gene annotations between neurons.
