# Supplementary material for: Hub connectivity, neuronal diversity, and gene expression in the Caenorhabditis elegans connectome
Source: PLoS Comput Biol. 2018 Feb 12;14(2):e1005989. doi: 10.1371/journal.pcbi.1005989 (PMC5825174; doi:10.1371/journal.pcbi.1005989)
Supplement: S3 Text — (PDF) [file pcbi.1005989.s003.pdf]

Correlated gene expression matching index

Existing measures of binary correlation (described below) are symmetric between '0' and '1' and thus do not directly distinguish between the biologically relevant case of genes being expressed together from the case in which neither gene is expressed. We thus developed a measure of the probability, *P(m)*, that two binary strings will contain *m* 'positive matches' (i.e., *m* genes are expressed in both neurons), which can be computed as:

$$P(m) = \binom{n_2}{m}\binom{N - n_2}{n_1 - m} / \binom{N}{n_1}$$

for two binary expression vectors, x_i, y_i, of length N containing n_1 and n_2 1s, respectively ($n_2 \leq n_2$), with m matches (n_{11}). Our index, p_{match}, is approximately equal to the probability of obtaining as many or fewer matches than observed (weighting the probability of the observed number of matches at 0.5 for symmetry), computed as:

$$p_{match} = \sum_{x=0}^{m-1} P(x) + P(m)/2$$

for m positive matches.

We verified that this index yields qualitatively similar results to the mean square contingency coefficient, r_ϕ, (S3 Fig), validating our related positive match method for scoring individual genes [as an approximately single-gene contribution to the probability score computed in Eq. (6)]

Given the qualitative similarity of this measure to r_ϕ, we chose to focus on r_ϕ throughout this work due to its ease of interpretability as a correlation coefficient ranging from -1 to 1.
