# Supplementary material for: Hub connectivity, neuronal diversity, and gene expression in the Caenorhabditis elegans connectome
Source: PLoS Comput Biol. 2018 Feb 12;14(2):e1005989. doi: 10.1371/journal.pcbi.1005989 (PMC5825174; doi:10.1371/journal.pcbi.1005989)
Supplement: S1 Table — Hubs are defined as neurons with degree k > 44. For each hub, we list (i) the neuron name, (ii) its degree, k, (iii) location (‘head’, ‘body’, or ‘tail’), and function based on information presented in the wormatlas website http://www.wormatlas.org/. Neurons are sorted (descending) by degree. (PDF) [file pcbi.1005989.s004.pdf]

**Neuron**	**Degree, k**	**Description**
AVAR	137	Head command interneuron, role in locomotor decisions
AVAL	134	Head command interneuron, role in locomotor decisions
AVBR	104	Head command interneuron, role in locomotor decisions
AVBL	102	Head command interneuron, role in locomotor decisions
PVCR	69	Tail command interneuron, role in locomotor decisions
PVCL	64	Tail command interneuron, role in locomotor decisions
AVDR	63	Head command interneuron, role in locomotor decisions
AVER	63	Head command interneuron, role in locomotor decisions
AVEL	62	Head command interneuron, role in locomotor decisions
DVA	59	Tail interneuron, mechanosensory integration
RIBL	56	Head interneuron
AVKL	53	Head interneuron
AVDL	52	Head command interneuron, role in locomotor decisions
RIBR	52	Head interneuron
AIBR	49	Head interneuron
RIGL	49	Head interneuron
