# Supplementary material for: Hub connectivity, neuronal diversity, and gene expression in the Caenorhabditis elegans connectome
Source: PLoS Comput Biol. 2018 Feb 12;14(2):e1005989. doi: 10.1371/journal.pcbi.1005989 (PMC5825174; doi:10.1371/journal.pcbi.1005989)
Supplement: S2 Table — Top 15 biological process GO categories enriched in genes with the highest mean increase in CGE for connected neurons compared to unconnected neurons. Categories are sorted by p-value (ascending). (PDF) [file pcbi.1005989.s005.pdf]

Category	Description	# genes	p (uncorr)	p (corr)
GO:0035235	ionotropic glutamate receptor signaling pathway	7	0.0005	0.3245
GO:0007215	glutamate receptor signaling pathway	9	0.0041	0.9367
GO:0007166	cell surface receptor signaling pathway	37	0.0048	0.9367
GO:0006811	ion transport	71	0.0058	0.9367
GO:0034220	ion transmembrane transport	57	0.0079	1
GO:0055085	transmembrane transport	67	0.0129	1
GO:1901575	organic substance catabolic process	33	0.031	1
GO:0040009	regulation of growth rate	7	0.0417	1
GO:0040010	positive regulation of growth rate	7	0.0417	1
GO:0030163	protein catabolic process	27	0.0428	1
GO:0009056	catabolic process	35	0.0493	1
GO:0009057	macromolecule catabolic process	28	0.0548	1
GO:0071495	cellular response to endogenous stimulus	14	0.0575	1
GO:0045927	positive regulation of growth	29	0.0689	1
GO:0050830	defense response to Gram-positive bakterium	5	0.0695	1
