# Supplementary material for: Hub connectivity, neuronal diversity, and gene expression in the Caenorhabditis elegans connectome
Source: PLoS Comput Biol. 2018 Feb 12;14(2):e1005989. doi: 10.1371/journal.pcbi.1005989 (PMC5825174; doi:10.1371/journal.pcbi.1005989)
Supplement: S3 Table — Top 15 biological process GO categories enriched in genes with the highest increase in CGE for connections involving hub neurons (i.e., rich, feed-in and feed-out connections) compared to connections between nonhub neurons (i.e., in peripheral connections). Categories are sorted by p-value (ascending). (PDF) [file pcbi.1005989.s006.pdf]

Category	Description	# genes	p (uncorr)	p (corr)
GO:0007215	glutamate receptor signaling pathway	6	0.0009	0.1741
GO:0035235	ionotropic glutamate receptor signaling pathway	6	0.0009	0.1741
GO:0007166	cell surface receptor signaling pathway	20	0.0035	0.3473
GO:0009891	positive regulation of biosynthetic process	11	0.0063	0.3473
GO:0031328	positive regulation of cellular biosynthetic process	11	0.0063	0.3473
GO:0045935	positive regulation of nucleobase-containing compound metabolic process	11	0.0063	0.3473
GO:0051173	positive regulation of nitrogen compound metabolic process	11	0.0063	0.3473
GO:0031325	positive regulation of cellular metabolic process	12	0.0109	0.5258
GO:0040012	regulation of locomotion	24	0.0156	0.5467
GO:0010557	positive regulation of macromolecule biosynthetic process	10	0.0212	0.5467
GO:0045893	positive regulation of transcription DNA-templated	10	0.0212	0.5467
GO:0045944	positive regulation of transcription from RNA polymerase II promoter	10	0.0212	0.5467
GO:0051254	positive regulation of RNA metabolic process	10	0.0212	0.5467
GO:1902680	positive regulation of RNA biosynthetic process	10	0.0212	0.5467
GO:1903508	positive regulation of nucleic acid-templated transcription	10	0.0212	0.5467
