# Supplementary figures and images for: Hub connectivity, neuronal diversity, and gene expression in the Caenorhabditis elegans connectome
Source: PLoS Comput Biol. 2018 Feb 12;14(2):e1005989. doi: 10.1371/journal.pcbi.1005989 (PMC5825174; doi:10.1371/journal.pcbi.1005989)

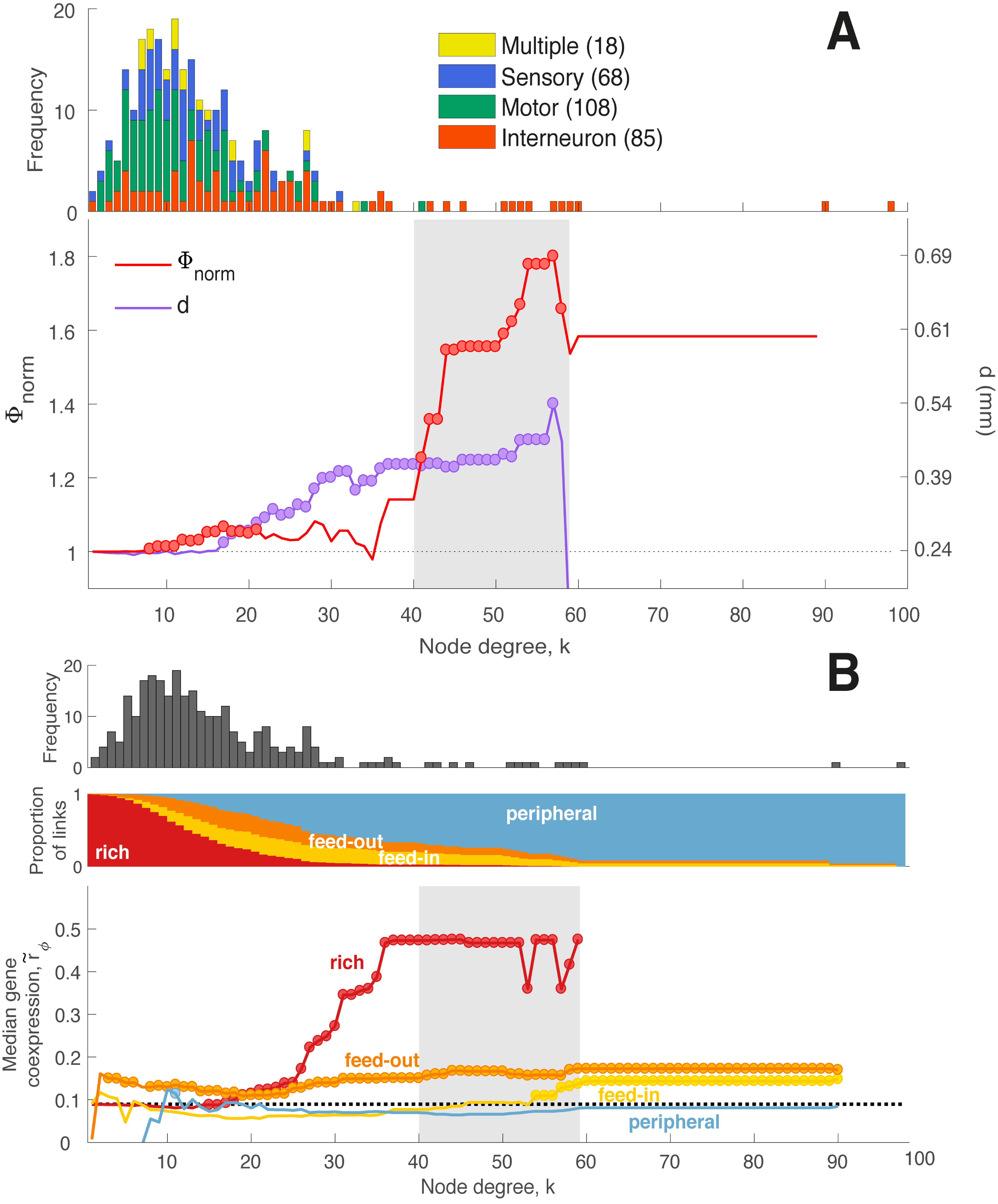

Supplement: S1 Fig — (A) Rich-club organization of the synaptic C. elegans connectome. Top: Degree distribution of neurons, labelled to four categories: (i) interneuron (85 neurons, orange), (ii) motor (108 neurons, green), (iii) sensory (68 neurons, blue), or (iv) multiple assignments (18 neurons, yellow). The distribution features an extended tail of high-degree neurons. Bottom: Normalized rich club coefficient, Φnorm (red), as a function of the degree, k, at which hubs are defined (as neurons with degree > k). Also shown is the mean Euclidean separation distance, d (purple) between connected hub regions (across degree thresholds, k). Φnorm > 1 indicates that hubs are more densely interconnected among each other than expected by chance, with red circles indicating values of Φnorm that are significantly higher than an ensemble of 1 000 degree-matched null networks (p < 0.05). Purple circles indicate where the Euclidean distance between connected pairs of hubs is significantly greater than the Euclidean distance for all other pairs of connected regions (right-tailed Welch’s t-test, p < 0.05). (B) Top: Degree distribution, k, of the synaptic C. elegans connectome. Middle: proportion of connections that are: ‘rich’ (hub → hub, red), ‘feed-in’ (nonhub → hub, yellow), ‘feed-out’ (hub → nonhub, orange), or ‘peripheral’ (nonhub → nonhub, blue) as a function of the degree threshold, k, used to define hubs. Note that at high k most neurons are labeled as nonhubs and hence the vast majority of connections are labeled ‘peripheral’. Bottom: Median CGE, r˜ϕ, for each connection type as a function of k. The median CGE across all network links is shown as a dotted black line; the topological rich-club regime (determined from the network topology, cf. A) is shaded gray. Circles indicate a statistically significant increase in CGE in a given link type relative to the rest of the network (one-sided Wilcoxon rank-sum test, p < 0.05). (TIF) [file pcbi.1005989.s007.tif]

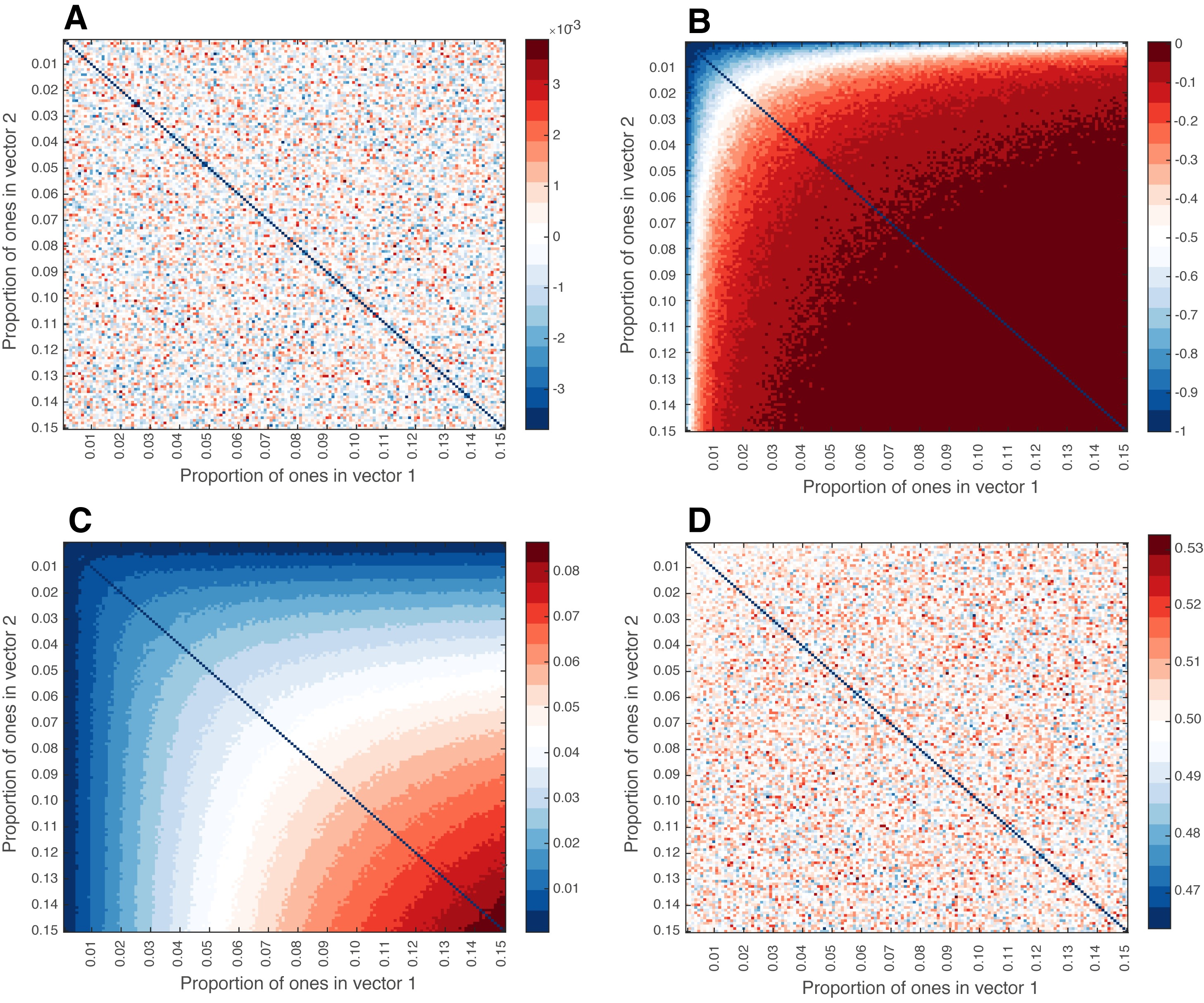

Supplement: S2 Fig — We plot the mean value of each metric across 1000 different pairs of random, binary vectors of length 948, which vary only in their proportion of ‘1’s (between 0–0.15; corresponding to a number of ‘1’s ranging from 1 to 150). This is repeated for: (A) mean square contingency coefficient, rϕ, (B) Jaccard index, (C) Yule’s Q, and (D) our developed positive match measure, pmatch, (see S3 Text). Any systematic trend in correlation values indicates a bias driven by the proportion of positive annotations for a pair of vectors, as is seen for the Jaccard index and Yule’s Q. By contrast, rϕ, which is used through this work, and our probability-based measure, pmatch, used to motivate individual gene scoring for enrichment analysis, show no evidence of systematic bias (note the color axis scales). (TIF) [file pcbi.1005989.s008.tif]

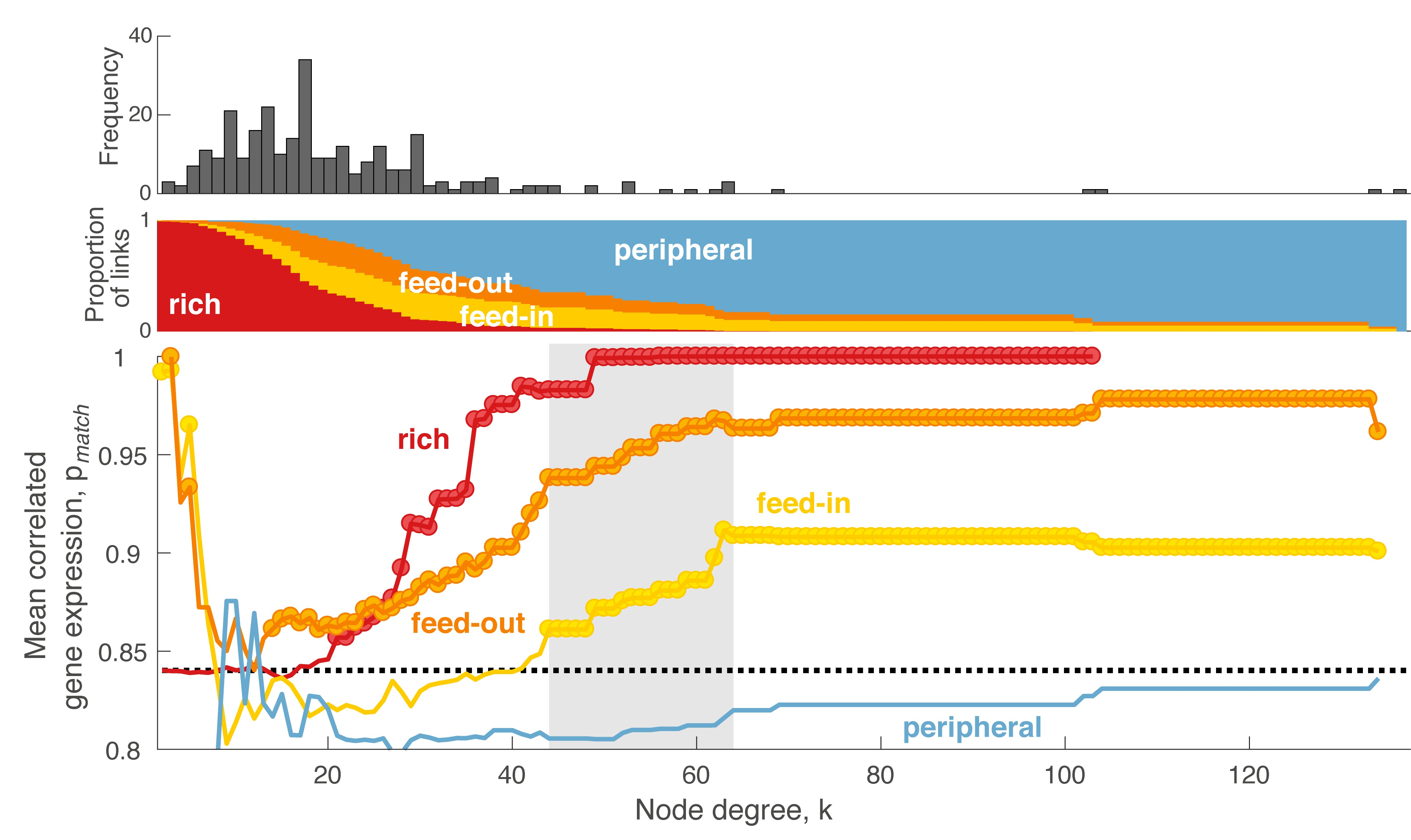

Supplement: S3 Fig — The matching probability index, pmatch, as introduced in S3 Text. Top: Degree distribution. Middle: Proportion of connections that are ‘rich’ (hub→hub, red), ‘feed-in’ (nonhub→hub, yellow), ‘feed-out’ (hub→nonhub, orange), and ‘peripheral’ (nonhub→nonhub, blue) as a function of the degree threshold, k, used to define hubs. Note that at high k, most neurons are labeled as nonhubs, and hence the vast majority of connections are ‘peripheral’. Bottom: Mean CGE calculated using similarity index from only positive matches, pmatch, for each connection type as a function of k. The mean CGE across all network links shown as a dotted black line; the topological rich-club regime (determined from the network topology, cf. Fig 5) is shaded gray. Circles indicate a statistically significant increase in CGE in a given link type relative to the rest of the network (one-sided Welch’s t test; p < 0.05). (TIF) [file pcbi.1005989.s009.tif]

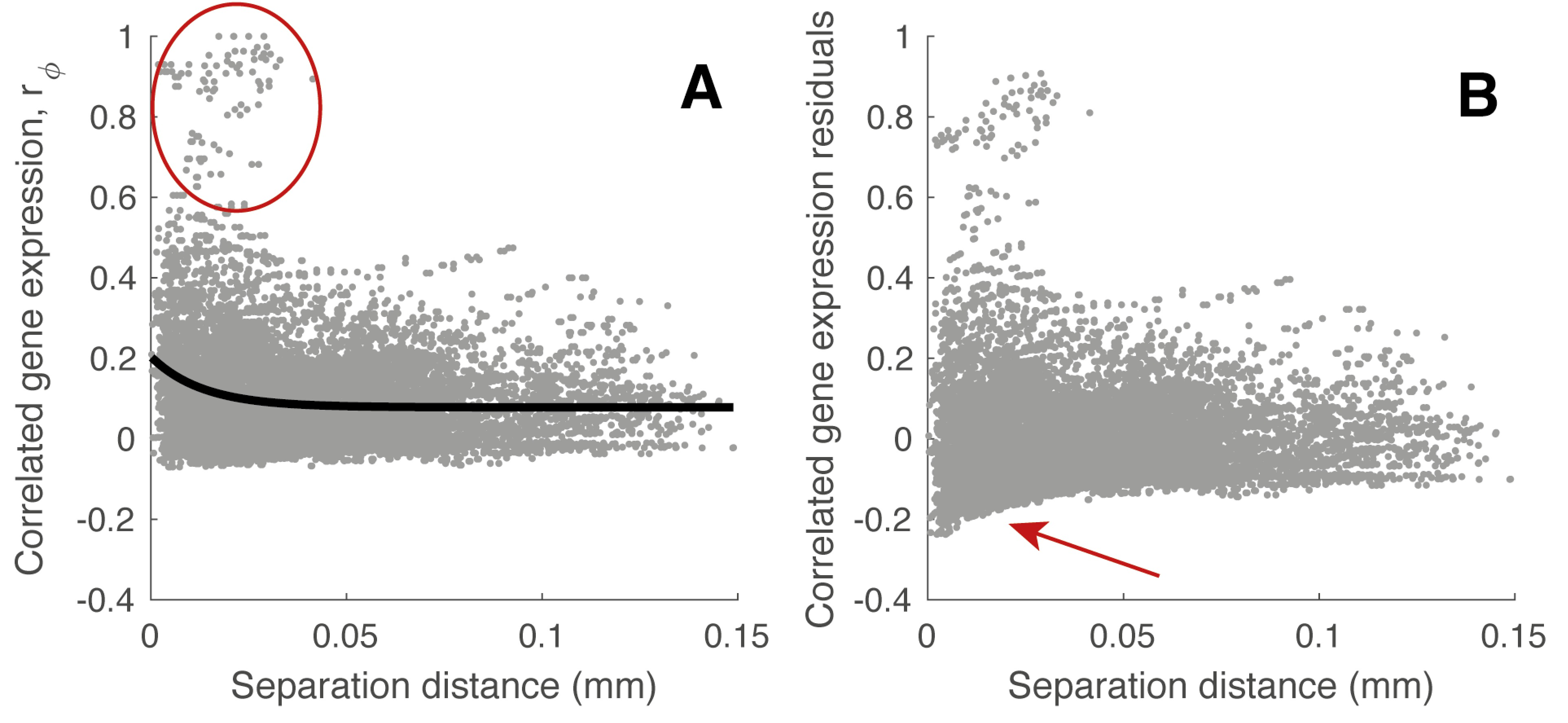

Supplement: S4 Fig — Here we consider correlated gene expression in the head, where the strongest spatial relationship exists (cf. Fig 4. (A) CGE values, rϕ, plotted as a function of Euclidean separation distance for all pairs of neurons within the head (gray dots), with a fitted exponential trend shown in black, f(x) = A exp(−λx) + B. (B) Taking residuals from this trend does not adequately correct the spatial trend. Note the artifactual negative correlations indicated with an arrow. This indicates that the trend is not a bulk, isotropic effect, but may instead be driven primarily by a small number of neuron pairs with high rϕ at short distances (⪅ 50μm), indicated with a circle in (A). For example, neuron pairs with rϕ > 0.8, are all between the following classes of head neurons: CEP, IL1, OLQ, RMD, RME, RMF, SAAD, SAAV, SAB, SIA, SIB, SMB, SMD, URA, URY. (TIF) [file pcbi.1005989.s010.tif]

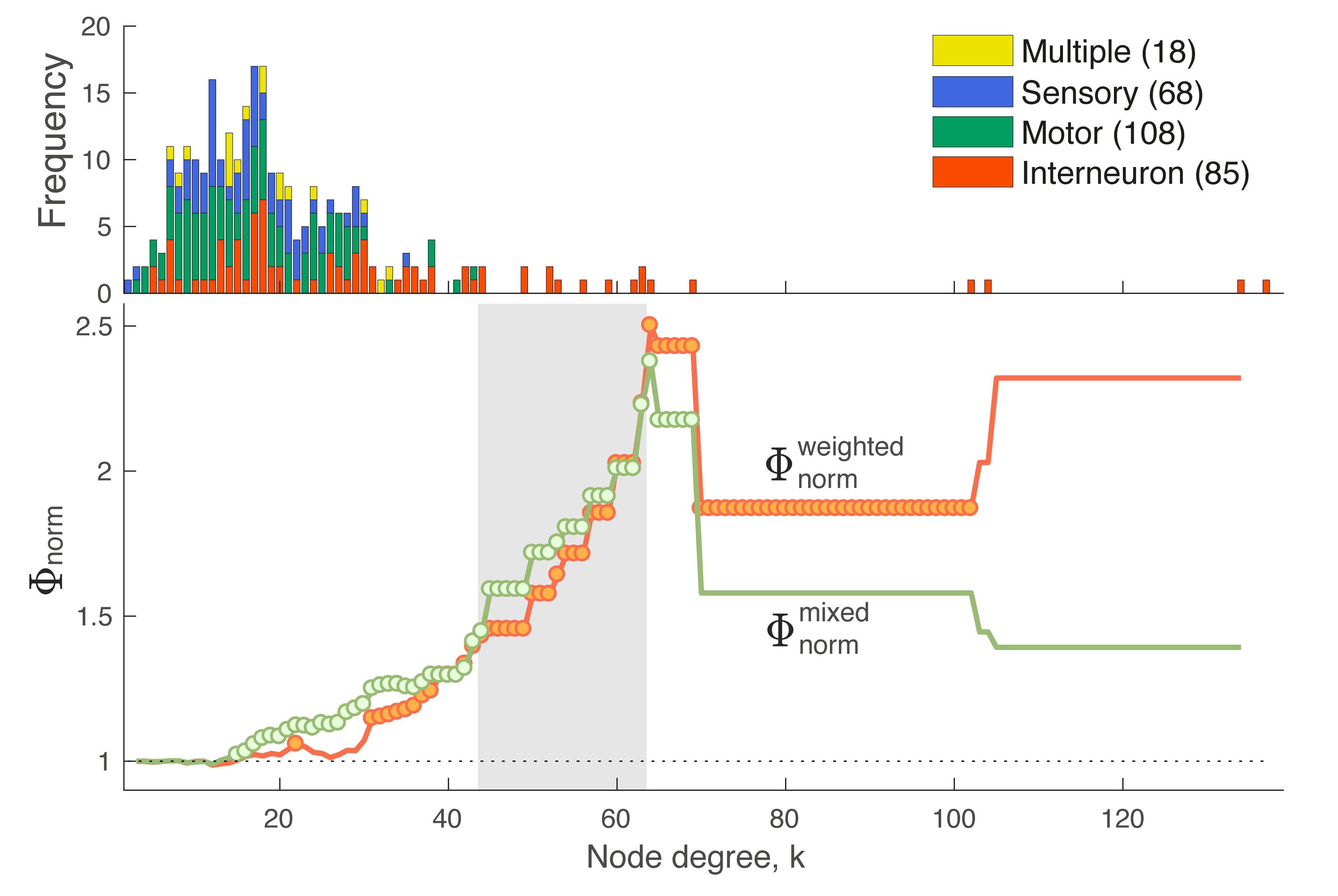

Supplement: S5 Fig — (A) Degree distribution of the C. elegans connectome. Neurons are labeled to four types as in the legend. (B) Normalized weighted rich-club coefficient, Φnormweighted (i.e., topology fixed and weights randomized in the null model, shown orange), and normalized mixed rich-club coefficient, Φnormmixed (i.e., both topology and weights mixed in the null model, shown green) are plotted as a function of the degree, k, at which hubs are defined (as neurons with degree > k) [129]. Circles indicate values of Φnorm that are significantly higher than an ensemble of 1 000 degree-matched null networks (Welch’s t-test, p < 0.05). Compared to topological rich-club analysis presented in the main text, here the weights of the connections are also accounted for when calculating the rich club coefficient. In the case of the weighted rich-club coefficient, the topology for the null models was kept stable and only the weights of the connections randomized. Results presented here show that connections between higher degree nodes are stronger than expected by chance. On the other hand, in the mixed rich club coefficient both the topology and weights are randomized, therefore we see the combined effect of both types. Distinction between the different null models is discussed in detail in [129]. These results show that connections between high degree nodes are both denser and stronger than expected by chance. (TIF) [file pcbi.1005989.s011.tif]

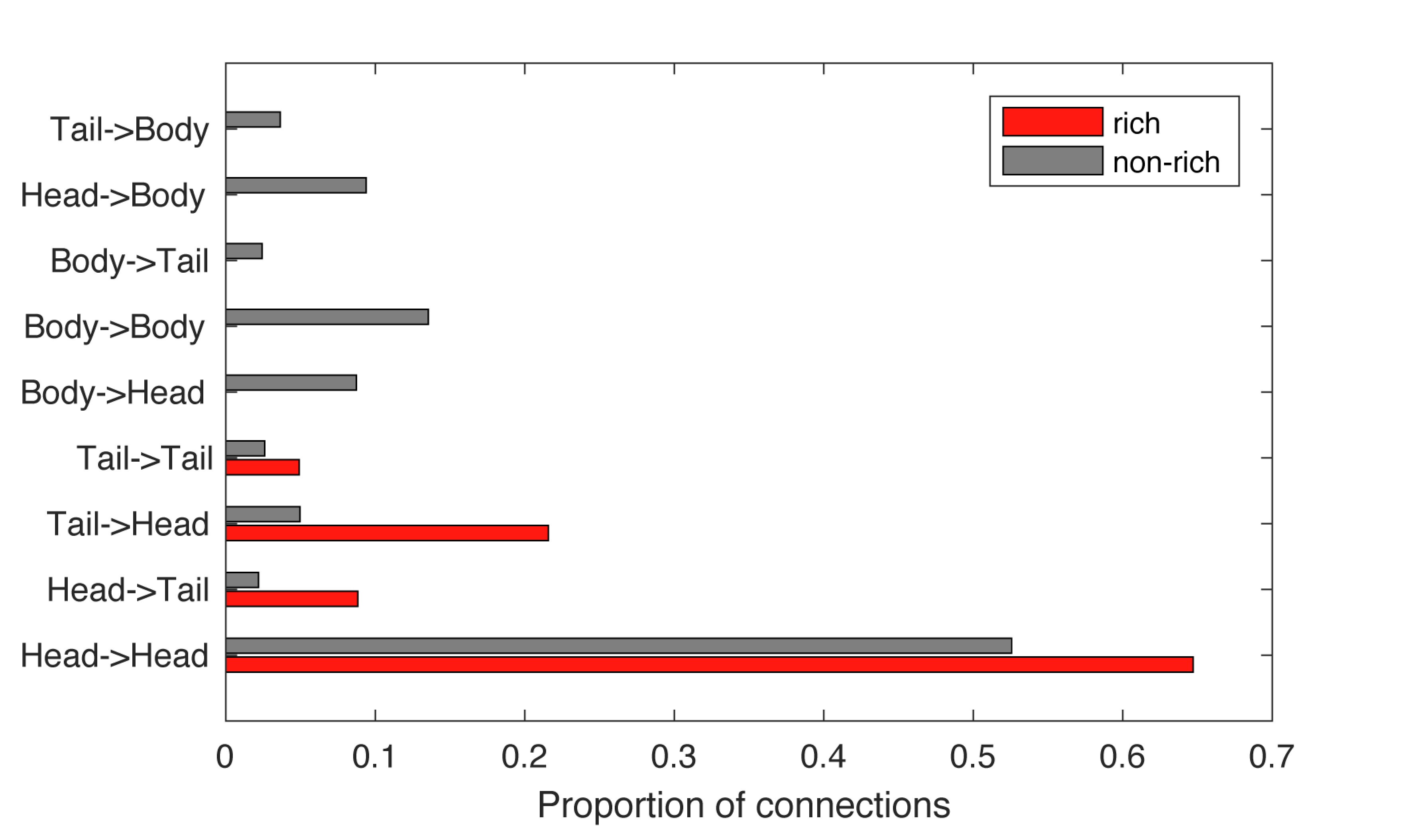

Supplement: S6 Fig — Hub-hub connections (‘rich’) are shown red, and all other connections (‘non-rich’, i.e., feeder and peripheral) are shown gray, where hubs are defined as neurons with degree, k > 44. Anatomical locations are labeled as ‘head’, ‘body’, and ‘tail’, and each connection is labeled according to its source and target neurons, listed on the vertical axis in the form ‘Source-Target’. The plot shows that the increased separation distance between connected hubs relative to other types of connected neurons is driven by a relative increase in long-range connections between the head and tail. (TIF) [file pcbi.1005989.s012.tif]
